# Development of Debonding-Resistant SBS–Silane Warm Mix Asphalt for Hot and Humid Pavement Conditions with Poor Aggregate Gradation

**DOI:** 10.3390/polym18121443

**Published:** 2026-06-09

**Authors:** Byung-Sik Ohm, Carlo Elipse, Tri Ho Minh Le

**Affiliations:** 1 Department of Highway & Transportation Research, Korea Institute of Civil Engineering and Building Technology, 283 Goyang-daero, Ilsanseo-gu, Goyang-si 10223, Republic of Korea; bseom@kict.re.kr (B.-S.O.); carloelipse@kict.re.kr (C.E.); 2 Faculty of Civil Engineering, Nguyen Tat Thanh University, 300A Nguyen Tat Thanh Street, District 4, Ho Chi Minh City 70000, Vietnam

**Keywords:** warm mix asphalt, SBS polymer modification, silane anti-stripping additive, moisture-induced debonding, Hamburg Wheel Tracking, fracture resistance

## Abstract

Asphalt pavements in hot and humid regions such as Southeast Asia are highly susceptible to moisture-induced debonding, especially when WMA is produced using marginal aggregates or less favorable gradation conditions. This study develops an anti-stripping-focused polymer-modified WMA system using SBS and a silane-based liquid additive. This study focuses on evaluating the coupled contribution of SBS-related binder cohesion and silane-related interfacial adhesion under poor gradation conditions, and verifies the selected system through binder-level, mixture-level, durability, and cost-efficiency evaluations. SBS contents of 4.0%, 4.5%, and 5.0% by binder mass were combined with silane dosages of 0%, 0.05%, 0.10%, and 0.15%. The mixtures were evaluated using MSCR, Marshall stability and flow, dry and wet ITS, TSR, Hamburg Wheel Tracking, SCB, and Overlay Test. SBS alone increased dry ITS and Marshall stability, but silane-free mixtures still showed low TSR values of 71.7–73.3%. The optimum mixture, S4.5-Si0.10, achieved a dry ITS of 0.94 MPa, wet ITS of 0.80 MPa, TSR of 85.1%, and Marshall stability of 13.8 kN. MSCR results confirmed that SBS reduced accumulated strain at both 0.1 and 3.2 kPa, while silane did not adversely affect binder deformation resistance. In Stage 2, the optimized SBS–silane mixture under poor gradation reduced Hamburg final settlement by 54.7% compared with the poor-gradation control. SCB work of fracture increased from 1.34 J to 5.20 J, and Overlay Test results confirmed improved load retention. The optimized mixture also reduced the annualized cost index by 27.2%. These findings demonstrate that a balanced SBS–silane WMA system can improve debonding resistance and durability under hot and humid pavement conditions.

## 1. Introduction

Warm mix asphalt (WMA) has attracted increasing attention as a practical pavement technology because it allows asphalt mixtures to be produced and compacted at lower temperatures than conventional hot mix asphalt [1]. The reduction in production temperature can decrease fuel consumption, emissions, binder aging, and construction-related environmental burdens, while improving working conditions during paving [2]. These advantages are especially relevant for regions with rapid infrastructure development, where large volumes of asphalt mixtures are required for road, expressway, and airport pavement construction. In Southeast Asia, including Vietnam, the demand for pavement materials has increased strongly in recent years. At the same time, pavement construction is frequently conducted under hot and humid environmental conditions, with repeated rainfall, high surface temperatures, and rapid wet–dry cycles [3]. These conditions create a challenging service environment for asphalt mixtures and require WMA systems that provide not only lower-temperature production benefits, but also sufficient durability against moisture damage and deformation.

Despite the environmental and construction advantages of WMA, its performance can be sensitive to mixture design and material quality. The lower mixing temperature may reduce binder aging and improve sustainability, but it can also reduce binder fluidity and aggregate coating efficiency if the mixture is not properly designed [4]. This concern becomes more critical when aggregate quality is variable or when gradation control is poor. In many developing pavement markets, the rapid growth of road and airport construction can increase the use of marginal aggregate sources or less favorable aggregate gradations [5]. Poor aggregate packing, excessive fine fraction, weak particle interlock, or high absorption can increase air void connectivity and promote water infiltration. Under hot and humid conditions, this can accelerate asphalt–aggregate debonding, stripping, rutting, cracking, and raveling [6]. Therefore, WMA mixtures designed for Southeast Asian service conditions should be evaluated not only for temperature reduction and workability, but also for moisture-induced debonding resistance under marginal aggregate conditions.

Polymer modification is one of the most common strategies for improving the high-temperature performance of asphalt mixtures [3]. Styrene–butadiene–styrene (SBS) is widely used to improve binder elasticity, cohesion, rutting resistance, and recovery under repeated loading [7]. In WMA systems, SBS modification can help compensate for the potential reduction in stiffness and deformation resistance associated with lower production temperature [2,8]. However, SBS mainly modifies the binder phase and improves the internal cohesive response of the asphalt binder. It does not necessarily solve the interfacial bonding problem between the asphalt binder and aggregate, especially when water is present. As a result, SBS-modified mixtures can exhibit high dry strength or improved rutting resistance, while still remaining vulnerable to moisture-induced debonding when the aggregate surface condition or gradation is unfavorable.

Several previous studies have confirmed that moisture damage and asphalt–aggregate debonding remain critical durability problems in asphalt mixtures, particularly when warm-mix technology, polymer modification, recycled materials, or moisture-sensitive aggregates are used. Sarkar et al. [9] investigated warm-mix additives, anti-stripping agents, and graphene nanoplatelets in stone mastic asphalt and reported that warm-mix additives generally improved cracking and stripping resistance, while graphene modification improved adhesion-related binder properties but did not always satisfy the TSR acceptance threshold. Peng et al. [10] developed a compound anti-stripping additive based on dopamine methacrylamide and nano-silicon dioxide dispersion, showing improved asphalt–aggregate adhesion, water stability, and mixture performance, especially for granite aggregate. Khani Sanij et al. [11] studied glass-containing warm mix asphalt modified with Zycotherm and found that the additive improved moisture susceptibility, resilient modulus, and creep performance. Hamedi and Tahami [12] further showed that Zycosoil improved moisture damage resistance by increasing cohesion energy and reducing debonding energy. More recently, Lu et al. [13] reported that silane-based KH-550 improved the adhesion work of PE-wax-modified asphalt to limestone aggregate, while Elnaml et al. [8] confirmed that amine-based liquid anti-strip additives enhanced TSR and rutting resistance under moisture conditioning. Dan et al. [14] also demonstrated that composite anti-stripping agents and hydrated lime improved the water stability of granite asphalt mixtures, with surface free energy showing strong correlation with macroscopic moisture damage indicators. These studies indicate that anti-stripping modification should not be evaluated only by conventional strength tests, but also through adhesion, moisture conditioning, cracking, rutting, and durability-related indicators.

Although previous studies have shown that WMA performance can be improved by polymer modification or anti-stripping additives, the two mechanisms are often interpreted separately. SBS-related improvement is mainly associated with binder cohesion, elasticity, and high-temperature deformation resistance, whereas silane or other anti-stripping additives mainly target asphalt–aggregate adhesion and moisture resistance. However, under poor gradation, mixture durability is not governed by either mechanism alone, because aggregate packing and connected void structure can also accelerate moisture access and debonding. Therefore, it remains unclear how binder-phase reinforcement, interfacial adhesion, and gradation quality jointly control WMA durability under moisture-sensitive conditions.

This study develops and evaluates an SBS–silane modified WMA system for hot and humid pavement conditions with poor aggregate gradation. The central scientific hypothesis is that the durability of such mixtures is governed by the coupled interaction among binder-phase cohesion, asphalt–aggregate interfacial bonding, and gradation-controlled moisture access. SBS modification is expected to improve binder cohesion, elastic recovery, and resistance to permanent deformation; however, these binder-phase benefits may not be sufficient to prevent moisture-induced debonding when poor aggregate packing creates easier water pathways. Silane addition is expected to improve wet interfacial bonding and reduce strength loss after moisture exposure, but its effectiveness may also depend on the aggregate structure and connected void system. Therefore, this study does not treat polymer modification and anti-stripping treatment as separate solutions. Instead, it evaluates whether SBS reinforcement and silane-based adhesion improvement can work together to maintain balanced rutting, moisture, and cracking resistance under gradation-induced moisture sensitivity.

The experimental program was organized in two stages to evaluate the proposed SBS–silane WMA system from screening to performance verification. In Stage 1, SBS contents of 4.0%, 4.5%, and 5.0% were combined with silane dosages of 0%, 0.05%, 0.10%, and 0.15% to identify a balanced modifier combination. The screening was based on dry and wet ITS, TSR, Marshall stability and flow, and MSCR response, so that both mixture moisture resistance and binder deformation behavior could be considered. Selected systems were then compared under standard and poor gradation conditions to assess gradation sensitivity. In Stage 2, representative mixtures were further evaluated using Hamburg Wheel Tracking, SCB, Overlay Test, and a performance-adjusted cost-efficiency index. The objective was not to select the mixture with the highest strength alone, but to determine whether an optimized SBS–silane WMA system could maintain a balanced response in terms of rutting resistance, moisture durability, fracture tolerance, crack-movement resistance, and economic efficiency under poor gradation and hot–humid service conditions.

## 2. Materials and Methods

### 2.1. Materials

The experimental program used a conventional paving asphalt binder, SBS polymer, a silane-based anti-stripping additive, crushed aggregates, and mineral filler. The SBS polymer and silane-based additive were supplied through a Vietnam–South Korea technical collaboration with a chemical company specializing in asphalt modification products. These materials were selected to develop a polymer-modified WMA mixture for hot and humid service conditions, where rutting resistance and moisture-induced debonding are both critical. SBS was incorporated to improve binder elasticity and high-temperature deformation resistance, while the silane additive was introduced to enhance asphalt–aggregate adhesion at reduced WMA production temperature. Two aggregate gradations were designed to evaluate the influence of aggregate structure, including a standard dense gradation and a poor gradation representing less favorable particle packing.

#### 2.1.1. Binder

The base binder properties are given in Table 1. The binder represents a common paving-grade asphalt used for dense-graded asphalt mixtures. Its properties were used as the reference condition for evaluating the effect of SBS and silane modification.

SBS polymer was incorporated at 4.0%, 4.5%, and 5.0% by binder mass. These dosages were selected to evaluate whether increasing polymer content could improve the binder and mixture response without reducing the balance between workability, strength, and moisture resistance. The main properties of the SBS modifier are listed in Table 2.

A silane-based liquid additive was used as an anti-stripping agent. The additive was added at 0%, 0.05%, 0.10%, and 0.15% by binder mass. Because of its low dosage, the silane additive was expected to act mainly at the asphalt–aggregate interface rather than as a bulk rheological modifier. Its typical properties are shown in Table 3. The silane additive used in this study was an amino-functional organosilane-based liquid anti-stripping agent. Its active chemical composition was mainly based on alkoxy-silane functional groups with amine functionality, commonly represented by a general structure of R–Si(OR′)_3_, where the hydrolysable alkoxy groups can interact with polar mineral aggregate surfaces and the organic functional group improves compatibility with the asphalt binder. This composition allows the additive to improve wetting, coating, and moisture resistance at the asphalt–aggregate interface.

#### 2.1.2. Aggregates and Fillers

Crushed mineral aggregates were used for both gradation conditions. The standard gradation was designed to provide a stable aggregate skeleton, whereas the poor gradation was designed to represent weaker particle packing and higher susceptibility to moisture-induced debonding. The basic aggregate and filler properties are listed in Table 4.

**Figure 1:**
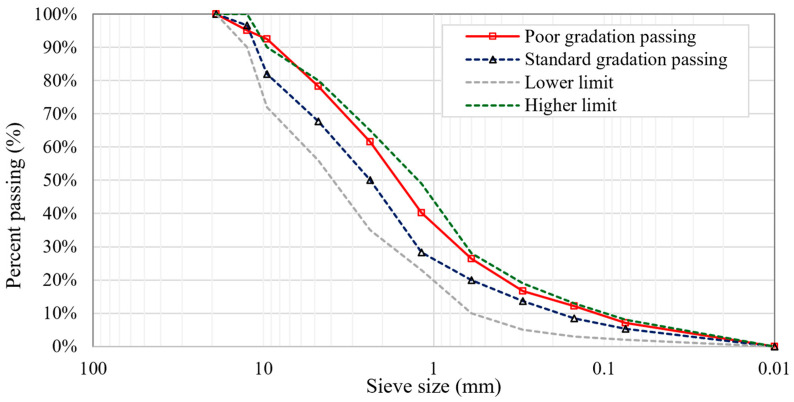
Sieve size analysis of the aggregate used in this research.

The aggregate gradations are presented in Figure 1. The poor gradation contained a lower passing percentage in the intermediate and fine sieve ranges, resulting in less favorable aggregate packing compared with the standard gradation. This allowed the study to evaluate whether the SBS–silane system could reduce the sensitivity of WMA mixtures to marginal gradation conditions.

It should be noted that the poor gradation was designed as a field-inspired, deliberately degraded aggregate structure rather than as a random gradation. In recent pavement maintenance and overlay projects in Vietnam, rapid infrastructure development has increased the demand for clean, well-graded aggregates. In some maintenance works, aggregate sources with less favorable particle packing, higher fine content, and less stable volumetric balance may be encountered. Field cores taken from conventional pavement and maintenance overlay sections showed that overlay mixtures produced with lower-quality aggregate tended to have higher air voids, lower VFA, higher dust-to-binder ratio, and a less favorable void structure. Therefore, the poor gradation used in this study was selected as a stress condition to represent a marginal aggregate structure that may increase moisture access and asphalt–aggregate debonding under hot and humid service conditions.

### 2.2. Mix Design

The binder systems are summarized in Table 5 and Table 6. The notation Sx-Siy was used, where x is the SBS content and y is the silane dosage. For example, S4.5-Si0.10 indicates a binder containing 4.5% SBS and 0.10% silane by binder mass.

The main mixture design parameters are given in Table 7. A fixed binder content was used for all mixtures to isolate the effects of SBS content, silane dosage, and gradation condition. The mixing and compaction temperatures were selected to represent WMA production.

The binder content of 5.4%, WMA mixing temperature of 135 °C, and compaction temperature of 125 °C were selected based on the authors’ previous research work on similar SBS-modified WMA mixtures and were verified through preliminary specimen preparation in this study [28,29]. These conditions provided acceptable aggregate coating, workability, and compactability for both standard and poor gradation mixtures. The same binder content and temperature conditions were therefore used for all mixtures to isolate the effects of SBS content, silane dosage, and gradation conditions.

This material system was designed to evaluate three coupled effects: SBS polymer reinforcement, silane-based interfacial adhesion improvement, and aggregate gradation quality. The combination of these variables allowed the study to assess whether a polymer-modified WMA system could maintain mechanical performance and moisture resistance under unfavorable aggregate conditions.

### 2.3. Mixing Process and Compaction Methods

The binder modification and mixture preparation procedures were designed to ensure consistent dispersion of SBS, uniform incorporation of the silane additive, and adequate aggregate coating at WMA production temperature (see Table 8). The process was divided into two parts: preparation of the modified binder and preparation/compaction of asphalt mixture specimens. The base asphalt binder was first heated to 165 ± 5 °C until it became fully fluid. SBS polymer was then added gradually at the target contents of 4.0%, 4.5%, and 5.0% by binder mass. The binder–polymer blend was mixed using a high-shear mixer at 3000 rpm for 45 min to promote polymer swelling and dispersion. After high-shear mixing, the binder was kept under low-speed stirring at 500 rpm for 20 min to improve homogeneity and reduce entrapped air. The silane additive was introduced after SBS modification at dosages of 0.05%, 0.10%, and 0.15% by binder mass. Because the silane dosage was low and its function was mainly interfacial, it was mixed at 500 rpm for 10 min at 150 ± 5 °C. The control binder and SBS-only binders were subjected to the same heating and stirring procedure to maintain comparable thermal histories.

The modified binder was held at approximately 150 °C for less than 2 h only to maintain workability before immediate mixture preparation. To check short-term homogeneity, representative SBS-modified binders were sampled from the upper and lower portions after the holding period, and the softening-point difference was found to be within about 1 °C. This indicates that no noticeable SBS phase separation occurred during the short laboratory holding period used in this study. However, this check was limited to short-term preparation conditions and does not represent long-term plant storage stability.

The asphalt mixtures were produced using a dense-graded WMA procedure. Aggregates and filler were oven-dried before mixing (see Table 9). The coarse and fine aggregate fractions were heated separately to 145 ± 5 °C, while the modified binder was maintained at 135 ± 5 °C before mixing. The selected WMA mixing temperature was lower than that commonly used for conventional hot mix asphalt, but sufficient for coating after SBS and silane modification. Dry aggregates were first mixed for 30 s to ensure uniform distribution. The binder was then added and mixed with the aggregates for 120 s. Mineral filler was introduced after the initial binder coating and mixed for an additional 60 s. The total wet mixing time was therefore approximately 180 s.

After mixing, the loose mixtures were short-term conditioned at 135 °C for 2 h to simulate plant production, transport, and placement before compaction. Specimens were compacted using a Superpave gyratory compactor and a Marshall compactor, depending on the target test. For ITS, TSR, SCB, OT, HWT, and dynamic modulus specimens, gyratory compaction was used to achieve the target air void level. For Marshall stability and flow, specimens were compacted using the Marshall hammer method. Table 10 presents the specimen compaction methods and target dimensions.

For Marshall specimens, the mixtures were compacted with 75 blows per side, which is commonly used for heavy-traffic dense-graded asphalt mixtures. For gyratory-compacted specimens, the number of gyrations was adjusted to reach the target air void content instead of using a fixed gyration number for all mixtures. This approach was used because SBS and silane modification can alter compactability and binder coating behavior at WMA temperature. After compaction, specimens were cooled at room temperature for 24 h before demolding or cutting. Table 11 presents the compaction and curing conditions.

The mixing and compaction procedure was kept constant for all mixtures so that the effects of SBS dosage, silane dosage, and gradation condition could be isolated. The use of reduced mixing and compaction temperatures was intended to represent WMA production, while the short-term conditioning step helped minimize differences caused by specimen preparation rather than material design. This procedure provided a consistent basis for comparing moisture susceptibility, rutting resistance, cracking resistance, and cost-efficiency among the selected mixtures.

### 2.4. Testing Methods

A multi-level testing program was conducted to evaluate the binder response, moisture susceptibility, rutting resistance, fracture behavior, and cyclic crack resistance of the WMA mixtures. Binder-level testing was first performed using the MSCR test to examine the stress-dependent deformation response of the control and modified binders. The Stage 1 mixture tests included Marshall stability, flow, dry ITS, wet ITS, and TSR to screen SBS and silane dosages and evaluate gradation sensitivity. The selected mixtures were then evaluated in Stage 2 using Hamburg Wheel Tracking, SCB, and Overlay Test. For each strength-based mixture test, at least three replicate specimens were tested, and the results were reported as mean values with standard deviations where applicable.

**Figure 2:**
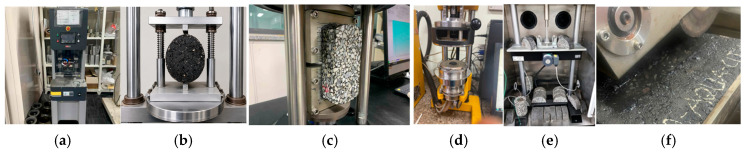
Laboratory testing procedures for asphalt mixture performance evaluation. (**a**) Compaction equipment, (**b**) IDT test, (**c**) OT test, (**d**) MSCR test, (**e**) SCB test setup, and (**f**) Hamburg wheel-tracking test.

The laboratory testing procedures used to evaluate the asphalt mixture performance are shown in Figure 2, including specimen compaction, IDT, OT, MSCR, SCB, and Hamburg wheel-tracking tests.

The Multiple Stress Creep Recovery test was conducted in accordance with ASTM D7405 [30] using a dynamic shear rheometer. The test was performed at two stress levels, 0.1 kPa and 3.2 kPa, to evaluate the deformation resistance and stress sensitivity of the binders. Each stress level consisted of 10 creep–recovery cycles, with 1 s of creep loading followed by 9 s of recovery. The accumulated strain response was recorded throughout the test. The non-recoverable creep compliance, $J_{nr}$, and recovery, R, were calculated as:

(1) $$J_{nr}=\frac{\varepsilon_{nr}}{\sigma }$$

(2) $$R=\frac{\varepsilon_{p}-\varepsilon_{nr}}{\varepsilon_{p}}\times 100$$

where $\varepsilon_{nr}$ is the non-recovered strain after the recovery period, σ is the applied stress, and $\varepsilon_{p}$ is the peak strain at the end of the creep loading stage.

Marshall stability and flow were measured in accordance with ASTM D6927 [31] to evaluate the basic load resistance and deformation response of the mixtures. Cylindrical specimens with a diameter of 101.6 mm and a height of approximately 63.5 mm were compacted using 75 blows per side. The specimens were conditioned at 60 °C before testing and loaded at a constant rate of 50.8 mm/min. Marshall stability was recorded as the maximum load, while flow was recorded as the deformation corresponding to the peak load.

The indirect tensile strength test was performed following ASTM D6931 [32], while the tensile strength ratio was determined following AASHTO T283 [33]. Cylindrical specimens with a diameter of 100 mm and a height of approximately 63.5 mm were prepared with a target air void content of 7.0 ± 0.5%. For each mixture, dry and moisture-conditioned specimens were prepared. The ITS test was conducted at 25 °C under a loading rate of 50 mm/min. The ITS and TSR were calculated using:

(3) $$ITS=\frac{2P}{\pi Dt}$$

(4) $$TSR=\frac{ITS_{wet}}{ITS_{dry}}\times 100$$

where P is the peak load, D is the specimen diameter, t is the specimen thickness, $ITS_{wet}$ is the average indirect tensile strength of moisture-conditioned specimens, and $ITS_{dry}$ is the average indirect tensile strength of dry specimens.

The Hamburg Wheel Tracking test was conducted in accordance with AASHTO T324 [34] to evaluate rutting resistance and moisture-related deformation under submerged loading. Compacted slab specimens with dimensions of approximately 320 × 260 × 60 mm were tested in a 50 °C water bath. The wheel load was applied up to 20,000 passes, and the wheel-path settlement was recorded during the test. The final settlement and settlement curve were used to compare rutting and stripping susceptibility among the selected mixtures.

The semi-circular bending test was used to evaluate fracture resistance and post-peak cracking behavior. Gyratory-compacted specimens were cut into semi-circular specimens with a diameter of 150 mm and a thickness of 50 mm. A notch depth of 15 mm was introduced at the center of the specimen before testing. The load–displacement response was recorded until the residual load became negligible. The work of fracture was calculated as the area under the load–displacement curve:

(5) $$W_{f}=\int P\;du$$

The fracture energy was then calculated as:

(6) $$G_{f}=\frac{W_{f}}{A_{lig}}\times 10^{6}$$

where $W_{f}$ is the work of fracture, P is the applied load, u is displacement, $A_{lig}$ is the ligament area, and $G_{f}$ is the fracture energy. In this study, P was expressed in kN and u in mm; therefore, 1kN\cdotpmm=1 J. The ligament area was calculated as the specimen thickness multiplied by the remaining ligament length.

The Overlay Test was conducted following Tex-248-F [35] to evaluate resistance to repeated crack movement. Beam specimens with dimensions of 150 × 75 × 38 mm were tested at 25 °C under repeated opening–closing displacement. The opening displacement was fixed at 0.635 mm, and the normalized peak load was recorded as a function of loading cycles. The normalized load ratio was calculated as:

(7) $$NLR_{i}=\frac{P_{i}}{P_{0}}$$

where $NLR_{i}$ is the normalized load ratio at cycle i, $P_{i}$ is the peak load at cycle i, and $P_{0}$ is the initial peak load.

A scenario-based performance-adjusted cost-efficiency index was also calculated to compare the selected mixtures after accounting for laboratory durability performance. This calculation was not intended to represent a project-specific life-cycle cost analysis. The performance index was calculated using the final Hamburg settlement, SCB work of fracture, and OT residual load at 1000 cycles:

(8) $$PI_{i}=0.40PI_{HWT,i}+0.30PI_{SCB,i}+0.30PI_{OT,i}$$

where

(9) $$PI_{HWT,i}=\frac{|HWT_{ref}|}{|HWT_{i}|}$$

(10) $$PI_{SCB,i}=\frac{W_{f,i}}{W_{f,ref}}$$

(11) $$PI_{OT,i}=\frac{OT_{i}}{OT_{ref}}$$

Because laboratory performance improvement cannot be directly converted into field service life, a conservative scenario-based coefficient was used in this study. The coefficient of 0.35 means that only 35% of the laboratory performance improvement was assumed to contribute to the relative service-life factor, based on the authors’ previous experience with pavement performance interpretation. This assumption was used to avoid overestimating the benefit of improved laboratory performance. Therefore, the calculated annualized cost index should be interpreted only as a relative cost-efficiency comparison among the tested mixtures, not as a field-calibrated life-cycle cost result.

(12) $$SLF_{i}=1+0.35(PI_{i}-1)$$

where $PI_{i}$ is the performance index and $SLF_{i}$ is the relative service-life factor. The coefficient 0.35 is a conservative scenario coefficient used to convert laboratory performance improvement into a relative service-life factor. Field performance monitoring is needed to calibrate this coefficient for actual pavement service-life prediction.

(13) $$ACI_{i}=\frac{C_{i}}{SLF_{i}}$$

where $C_{i}$ is the initial cost index and $ACI_{i}$ is the annualized cost index. The poor-gradation control mixture was used as the reference condition for the index calculation.

To further verify durability after environmental conditioning, the two optimized mixtures, S4.5-Si0.10-Gpoor and S4.5-Si0.10-Gstd, were subjected to 10 freeze–thaw cycles before repeated HWT and SCB testing. After compaction, all specimens were cured at room temperature for 24 h. Each freeze–thaw cycle consisted of freezing at −18 ± 2 °C for 6 h followed by thawing in a 25 ± 2 °C water bath for 6 h, giving a total cycle duration of 12 h. After completing 10 cycles, the specimens were surface-dried, equilibrated to the target test temperature, and then tested using the same HWT and SCB procedures described above.

All major mixture performance results were obtained from replicate specimens. The ITS, TSR, and Marshall test results are presented as mean values with corresponding error bars, and the experimental variability of the primary performance indicators was controlled within approximately 15% deviation.

One-way analysis of variance (ANOVA) was additionally conducted to verify the statistical significance of the main performance differences among the SBS–silane WMA mixtures at a 95% confidence level. For advanced performance tests, including HWT, SCB, and OT, the results are reported as representative mean responses, which is consistent with common practice in pavement materials studies due to the higher testing complexity, specimen preparation time, and limited number of replicate specimens.

**Figure 3:**
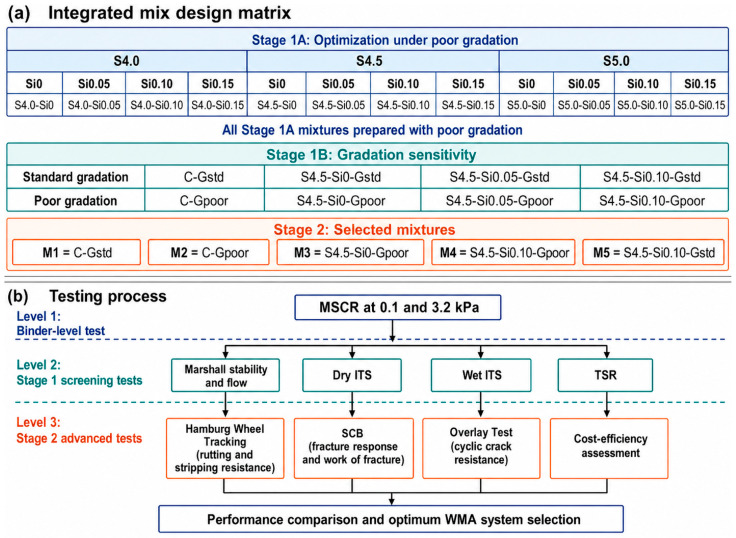
Integrated mix design matrix and testing process for the SBS–silane WMA experimental program. *S denotes SBS polymer content by binder mass; Si denotes silane additive content by binder mass; Gstd denotes standard gradation; Gpoor denotes poor gradation; C denotes the control WMA mixture without SBS and silane additive. For example, S4.5-Si0.10-Gpoor represents a WMA mixture containing 4.5% SBS, 0.10% silane additive, and poor aggregate gradation.*

The overall experimental framework is summarized in Figure 3. The mix design matrix was organized to progressively evaluate the proposed SBS–silane WMA system from dosage screening to performance verification. Stage 1A was used to optimize SBS and silane additive dosages under poor gradation conditions, while Stage 1B examined the sensitivity of selected mixtures to standard and poor aggregate gradations. Based on these screening results, five representative mixtures were selected for Stage 2 advanced testing, including Hamburg Wheel Tracking, SCB, Overlay Test, and performance-adjusted cost-efficiency assessment. This staged framework was designed to connect binder-level MSCR behavior with mixture-level ITS/TSR, rutting, cracking, and durability performance, thereby allowing the roles of SBS modification, silane-based anti-stripping treatment, and aggregate gradation to be evaluated systematically.

It should be noted that for the main strength-based screening tests, at least three replicate specimens were tested, and the results are presented as mean values with standard deviations/error bars. These include dry ITS, wet ITS, TSR, Marshall stability, and flow results. For MSCR, Hamburg Wheel Tracking, and SCB tests, the results are presented as response curves to show the full deformation or load–displacement behavior. Therefore, standard deviations were not plotted directly on these Figures to maintain curve readability; these tests are discussed based on representative response trends and key values such as accumulated strain, final settlement, peak load, and work of fracture.

## 3. Results and Discussion

### 3.1. Stage 1: Optimization of SBS and Silane Additive Contents Based on ITS and TSR

**Figure 4:**
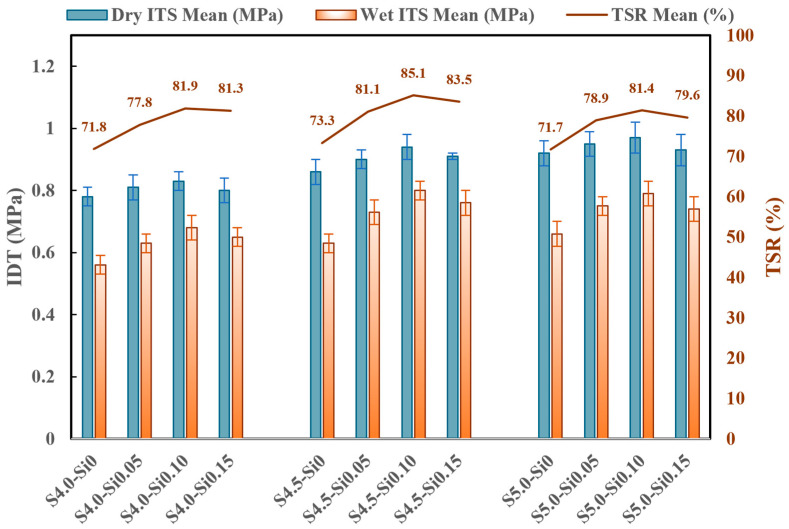
Dry ITS, wet ITS, and TSR results of SBS–silane WMA mixtures under poor gradation conditions.

The Stage 1 screening results are presented in Figure 4. This stage was used to identify a balanced SBS–silane combination that could improve tensile strength and moisture resistance under poor gradation conditions. For the silane-free mixtures, increasing SBS content improved dry ITS from 0.78 MPa for S4.0-Si0 to 0.86 MPa for S4.5-Si0 and 0.92 MPa for S5.0-Si0. However, the corresponding TSR values remained low at 71.7–73.3%, indicating that SBS mainly improved binder cohesion and tensile strength, but did not sufficiently prevent moisture-induced strength loss.

Silane addition clearly improved moisture resistance. At 4.0% SBS, TSR increased from 71.8% without silane to 77.8% at 0.05% silane and 81.9% at 0.10% silane. At 4.5% SBS, TSR increased from 73.3% to 81.1% and 85.1% at the same silane dosages. The best balance was obtained for S4.5-Si0.10, which achieved a dry ITS of 0.94 MPa, wet ITS of 0.80 MPa, and TSR of 85.1%. Compared with the SBS-only mixture at the same SBS content, the wet ITS increased from 0.63 MPa to 0.80 MPa and TSR increased by 11.8 percentage points, confirming that silane mainly improved wet strength retention through better asphalt–aggregate adhesion.

Increasing the silane dosage to 0.15% did not provide further improvement. For example, TSR decreased from 85.1% to 83.5% at 4.5% SBS and from 81.4% to 79.6% at 5.0% SBS when silane increased from 0.10% to 0.15%. Although the 5.0% SBS mixtures produced the highest dry ITS values, their TSR values were lower than those of the optimized 4.5% SBS system. This indicates that higher SBS or silane content does not necessarily improve moisture resistance and may reduce the balance between strength, coating efficiency, and interfacial response. Therefore, S4.5-Si0.10 was selected as the optimized SBS–silane WMA system for the next evaluation stage.

### 3.2. Stage 1: Marshall Stability and Flow Behavior

**Figure 5:**
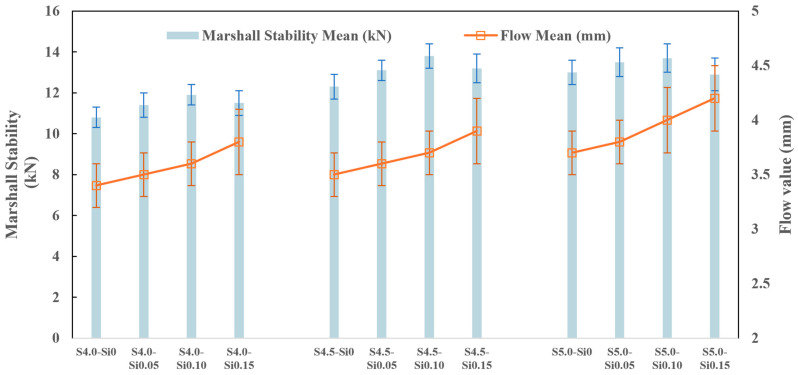
Marshall stability and flow results of SBS–silane WMA mixtures under poor gradation conditions.

The Marshall stability and flow results are shown in Figure 5. Overall, Marshall stability increased with SBS content, confirming the contribution of polymer modification to mixture cohesion and load resistance. For the silane-free mixtures, stability increased from 10.8 kN for S4.0-Si0 to 12.3 kN for S4.5-Si0 and 13.0 kN for S5.0-Si0. However, the corresponding flow also increased from 3.4 mm to 3.7 mm, indicating that higher SBS content changed the strength–deformation balance rather than simply increasing stiffness.

The addition of silane further improved stability, especially at the 0.10% dosage. At 4.0% SBS, stability increased from 10.8 kN to 11.9 kN, while at 4.5% SBS, it increased from 12.3 kN to 13.8 kN. The best Marshall response was obtained for S4.5-Si0.10, which achieved the highest stability of 13.8 kN while maintaining a moderate flow value of 3.7 mm. This agrees with the ITS and TSR results, suggesting that 0.10% silane improved the binder–aggregate interaction and contributed to a better strength–deformation balance under poor gradation.

Increasing the silane dosage to 0.15% did not provide further benefit. At 4.5% SBS, stability decreased from 13.8 kN to 13.2 kN, and flow increased from 3.7 mm to 3.9 mm. Similarly, at 5.0% SBS, stability decreased from 13.7 kN at 0.10% silane to 12.9 kN at 0.15% silane, while flow increased to 4.2 mm. These results suggest a possible overdosing effect, where excessive silane or polymer content may reduce the balance between coating, strength, and deformation response. Therefore, the Marshall results support the selection of S4.5-Si0.10 as the optimized mixture for subsequent evaluation.

### 3.3. Stage 1B: Effect of Gradation Condition on ITS and TSR

**Figure 6:**
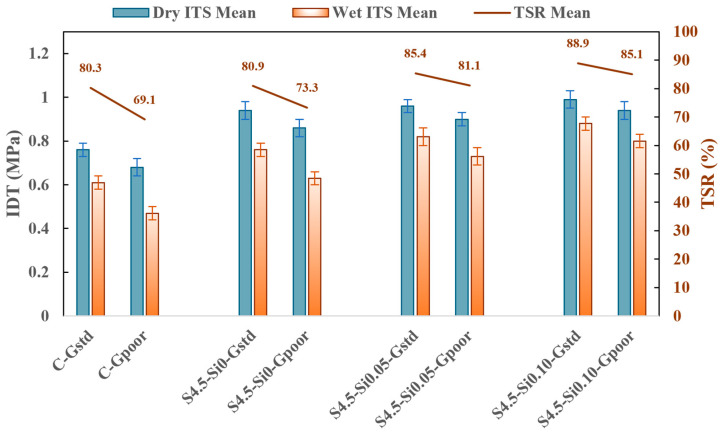
Effect of standard and poor gradation conditions on dry ITS, wet ITS, and TSR of selected SBS–silane WMA mixtures.

The gradation sensitivity results are shown in Figure 6. This stage evaluated whether the optimized SBS–silane system could maintain its effectiveness under both standard and poor gradation conditions. The control mixture was strongly affected by poor gradation: dry ITS decreased from 0.76 to 0.68 MPa, wet ITS decreased from 0.61 to 0.47 MPa, and TSR dropped from 80.3% to 69.1%. This confirms that poorer aggregate packing increased moisture sensitivity, likely by promoting less uniform binder film distribution, higher local void connectivity, and easier water intrusion.

SBS modification improved tensile strength but did not fully overcome moisture-induced strength loss. For S4.5-Si0, changing from standard to poor gradation reduced dry ITS from 0.94 to 0.86 MPa, wet ITS from 0.76 to 0.63 MPa, and TSR from 80.9% to 73.3%. This indicates that SBS enhanced binder cohesion and tensile resistance, but did not sufficiently stabilize the aggregate–binder interface under wet conditions.

The addition of silane improved wet strength retention more effectively. At 0.05% silane, TSR increased to 85.4% under standard gradation and 81.1% under poor gradation. The best response was obtained for S4.5-Si0.10, which achieved dry ITS/wet ITS/TSR values of 0.99 MPa/0.88 MPa/88.9% under standard gradation and 0.94 MPa/0.80 MPa/85.1% under poor gradation. A key finding is that the TSR loss caused by poor gradation decreased from 11.2 percentage points for the control mixture to 7.6 percentage points for the SBS-only mixture and only 3.8 percentage points for S4.5-Si0.10. This confirms that the optimized SBS–silane system reduced gradation sensitivity by combining binder cohesion improvement with stronger wet interfacial bonding.

These results support the selection of S4.5-Si0.10 as the optimized WMA system for advanced testing. S4.5-Si0.10-Gpoor was selected as the main proposed mixture for Stage 2 verification, while S4.5-Si0.10-Gstd was retained as the optimized standard-gradation reference.

### 3.4. Stage 1A: MSCR Response of SBS–Silane Modified Binders

**Figure 7:**
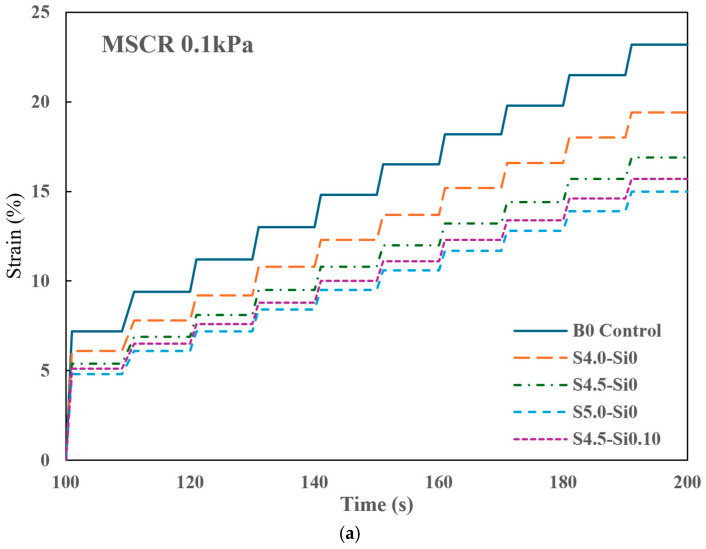

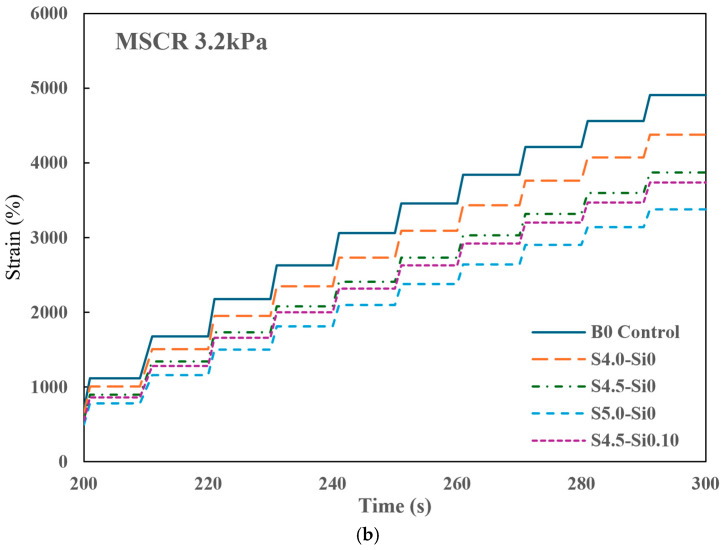
MSCR accumulated strain response of control and SBS–silane modified WMA binders at (**a**) 0.1 kPa and (**b**) 3.2 kPa.

The MSCR results at 0.1 kPa and 3.2 kPa are shown in Figure 7. The accumulated strain response clearly indicates that SBS modification improved the high-temperature deformation resistance of the WMA binder system. At 0.1 kPa, the control binder reached an accumulated strain of 23.2% at 200 s, whereas the SBS-modified binders showed lower final strain values. The accumulated strain decreased to 19.4% for S4.0-Si0, 16.9% for S4.5-Si0, and 15.0% for S5.0-Si0, corresponding to reductions of approximately 16.4%, 27.2%, and 35.3%, respectively, compared with the control binder. This trend confirms that increasing SBS content strengthened the elastic polymer network and reduced unrecovered strain during repeated creep–recovery loading.

A similar but more pronounced trend was observed at 3.2 kPa, where stress sensitivity became more evident. The control binder accumulated a final strain of 4910%, while S4.0-Si0, S4.5-Si0, and S5.0-Si0 decreased to 4380%, 3870%, and 3380%, respectively. Relative to the control binder, these reductions were approximately 10.8%, 21.2%, and 31.2%. The stronger separation among binders at 3.2 kPa indicates that the benefit of SBS modification became more important under higher stress conditions. This is consistent with the expected role of SBS in improving elastic recovery and reducing stress-dependent deformation in asphalt binders used for rutting-prone environments.

The binder containing both SBS and silane, S4.5-Si0.10, showed an accumulated strain of 15.7% at 0.1 kPa and 3740% at 3.2 kPa. These values were slightly lower than those of S4.5-Si0 at 3.2 kPa and close to the same binder at 0.1 kPa, indicating that the silane additive did not adversely affect the stress-dependent binder response. This point is important because silane was introduced mainly to improve aggregate–binder adhesion and moisture resistance, not to act as a primary rheological modifier. The MSCR results therefore confirm that the optimized SBS–silane binder retained adequate rutting resistance while providing the interfacial benefit observed in the ITS, TSR, and Marshall results.

Although S5.0-Si0 showed the lowest accumulated strain in both stress levels, this binder was not selected as the optimum system because the mixture-level results did not show proportional benefits at 5.0% SBS. The ITS, TSR, and Marshall results indicated that excessive SBS content could increase dry strength or reduce binder strain, but it did not necessarily improve moisture resistance or deformation balance at WMA production temperature. Therefore, the MSCR results should be interpreted together with the mixture performance results. From this perspective, S4.5-Si0.10 provided the most practical balance: it maintained strong rutting resistance at the binder level while achieving superior moisture resistance and mixture stability under poor gradation conditions.

### 3.5. Stage 2: Hamburg Wheel Tracking Performance

**Figure 8:**
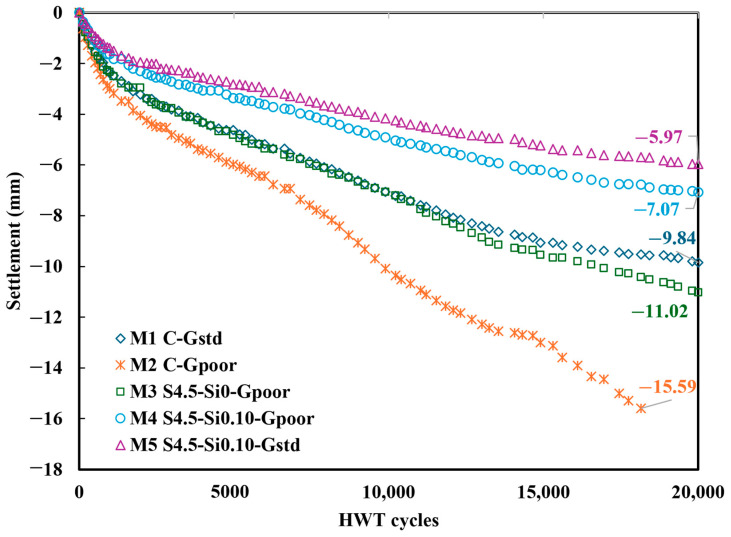
Hamburg Wheel Tracking settlement curves of selected WMA mixtures under submerged loading conditions.

The Hamburg Wheel Tracking results are shown in Figure 8. The test was used to verify the rutting and moisture-related deformation resistance of the selected WMA mixtures under submerged loading conditions. The results show a clear influence of gradation condition and binder–additive system on accumulated settlement. The poor-gradation control mixture, M2 C-Gpoor, exhibited the highest final settlement of −15.59 mm, indicating the weakest resistance to combined wheel loading and water exposure. In contrast, the standard-gradation control, M1 C-Gstd, reached a lower final settlement of −9.84 mm, corresponding to a reduction of approximately 36.9% compared with M2. This confirms that poor gradation substantially increased deformation and moisture-damage sensitivity, likely due to weaker aggregate interlock, less stable particle packing, and easier water penetration along interconnected void paths.

The SBS-only poor-gradation mixture, M3 S4.5-Si0-Gpoor, reduced the final settlement to −11.02 mm, which represents an improvement of approximately 29.3% compared with M2 C-Gpoor. This result shows that SBS modification improved high-temperature deformation resistance by strengthening the binder network and increasing mixture cohesion. Nevertheless, M3 still performed worse than M1 C-Gstd, even though it contained SBS. This indicates that polymer modification alone could not fully compensate for the unfavorable gradation condition. The curve of M3 also continued to accumulate deformation at a relatively high rate in the later loading stage, suggesting that the poor aggregate structure and moisture-induced weakening remained active during submerged wheel tracking.

The optimized SBS–silane mixture under poor gradation, M4 S4.5-Si0.10-Gpoor, showed a much better response, with final settlement limited to −7.07 mm. Compared with M2 C-Gpoor, this corresponds to a reduction of approximately 54.7%. Compared with the SBS-only poor-gradation mixture, M4 reduced settlement by approximately 35.8%. This improvement demonstrates that the silane additive provided additional benefit beyond SBS modification. While SBS mainly improved binder elasticity and rutting resistance, silane likely enhanced asphalt–aggregate adhesion and reduced moisture-induced debonding under water-loaded conditions. This mechanism is consistent with the Stage 1 ITS and TSR results, where silane improved wet strength retention and reduced the sensitivity of poor gradation mixtures to moisture damage.

The best HWT performance was obtained for M5 S4.5-Si0.10-Gstd, which reached a final settlement of only −5.97 mm. This value is approximately 61.7% lower than that of M2 C-Gpoor and 15.6% lower than that of M4 S4.5-Si0.10-Gpoor. The comparison between M4 and M5 confirms that the optimized SBS–silane binder system performed well under both gradation conditions, but the standard gradation still provided additional structural benefit through better aggregate packing and load distribution. The ranking of the mixtures followed the order M5 > M4 > M1 > M3 > M2, showing that the best performance required both interfacial improvement and a stable aggregate skeleton.

This discussion is also in line with recent sustainable WMA studies. For example, Abdulrahman et al. [36] showed that sustainable warm asphalt mixtures using construction and demolition waste aggregates should be evaluated in terms of both stripping resistance and cracking performance. This supports the broader interpretation of the present study that moisture resistance, fracture response, and aggregate structure should be considered together when developing durable WMA systems for severe service conditions.

In general, the HWT results support the proposed mechanism of this study. Poor gradation increased susceptibility to rutting and moisture-related settlement. SBS modification improved deformation resistance, but its benefit was limited when the mixture remained vulnerable to debonding. The combination of 4.5% SBS and 0.10% silane produced a more balanced WMA system by maintaining binder-level rutting resistance while strengthening the aggregate–binder interface. Therefore, S4.5-Si0.10-Gpoor can be considered an effective solution for WMA mixtures produced with marginal gradation or poor aggregate conditions in hot and humid regions.

### 3.6. Stage 2: SCB Fracture Response

**Figure 9:**
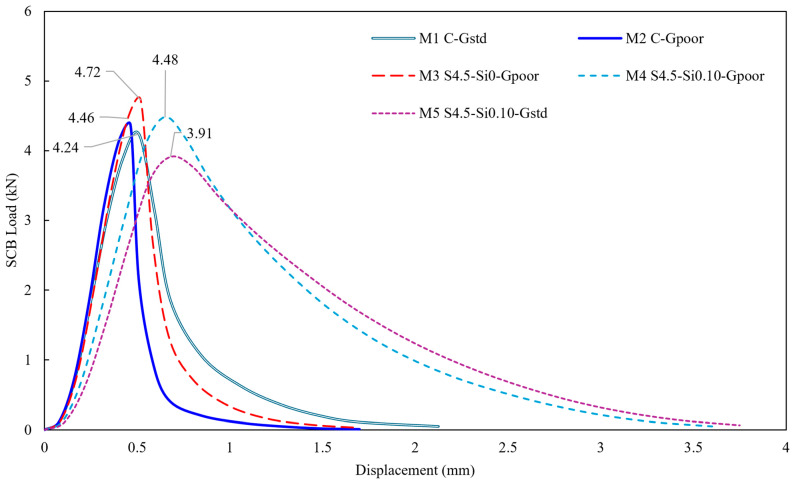
SCB load–displacement curves of selected WMA mixtures under standard and poor gradation conditions.

The SCB load–displacement responses of the selected WMA mixtures are shown in Figure 9. The curves show that the mixtures had clearly different fracture behaviors, especially in the post-peak region. Although peak load is commonly used as a strength indicator, the present results show that peak load alone does not fully describe cracking resistance. Some mixtures developed relatively high peak loads but lost their load-carrying capacity rapidly after cracking, whereas the optimized SBS–silane mixtures maintained load over a longer displacement range.

The poor-gradation control mixture, M2 C-Gpoor, exhibited a peak load of 4.36 kN at a displacement of 0.465 mm. However, its load dropped sharply to 2.12 kN at 0.510 mm and further decreased to 0.86 kN at 0.595 mm. This sudden post-peak reduction indicates brittle fracture behavior. Although the peak load of M2 was not the lowest, its post-peak resistance was poor, resulting in the lowest work of fracture of 1.34 J. This confirms that poor gradation reduced fracture tolerance by weakening aggregate interlock and accelerating crack propagation after peak loading.

**Figure 10:**
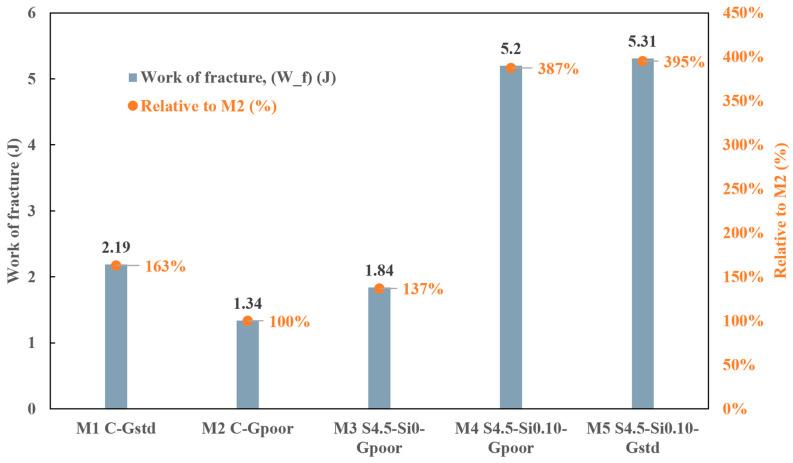
Work of fracture and relative fracture energy index of selected WMA mixtures based on SCB test results.

The SCB load–displacement results show clear differences in fracture behavior among the five mixtures. The poor-gradation control mixture, M2 C-Gpoor, had the weakest fracture response, with the lowest work of fracture of 1.34 J as shown in Figure 10. This confirms that poor gradation reduced the ability of the mixture to resist crack growth, likely because the less stable aggregate structure promoted weaker load transfer and easier crack propagation.

The SBS-only poor-gradation mixture, M3 S4.5-Si0-Gpoor, produced the highest peak load of 4.72 kN, indicating that SBS improved binder cohesion and initial load-bearing capacity. However, the post-peak load decreased rapidly, and the work of fracture reached only 1.84 J. This value was higher than M2 but still lower than the standard-gradation control mixture, M1 C-Gstd, which had a work of fracture of 2.19 J. Therefore, SBS alone improved peak resistance but did not sufficiently improve post-cracking tolerance when the mixture still had poor gradation and weak moisture-sensitive interfacial conditions.

In contrast, the optimized SBS–silane poor-gradation mixture, M4 S4.5-Si0.10-Gpoor, showed a more stable post-peak response. Although its peak load of 4.48 kN was slightly lower than that of M3, it maintained load over a wider displacement range and achieved a much higher work of fracture of 5.20 J. This indicates that silane addition improved fracture tolerance mainly by enhancing asphalt–aggregate bonding and delaying post-peak load loss, rather than simply increasing peak strength.

The optimized standard-gradation mixture, M5 S4.5-Si0.10-Gstd, showed the highest work of fracture of 5.31 J, despite having a lower peak load of 3.91 kN. This confirms that standard gradation further improved fracture resistance by providing a more stable aggregate skeleton and more gradual crack propagation. The fracture energy ranking was therefore M5 ≈ M4 > M1 > M3 > M2. Overall, the SCB results demonstrate that the optimized SBS–silane system improved cracking resistance primarily by increasing post-peak load retention and energy absorption, while the best performance was achieved when improved interfacial bonding was combined with standard aggregate gradation.

### 3.7. Stage 2: Overlay Test Response

**Figure 11:**
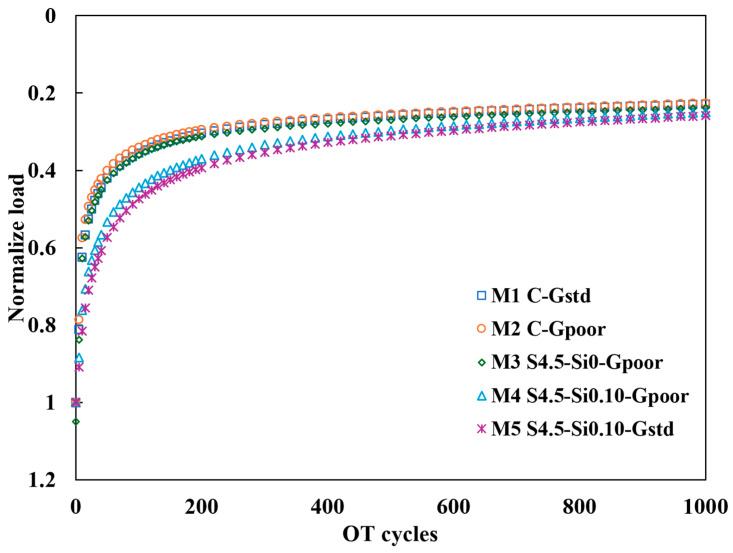
Overlay Test normalized load degradation curves of selected WMA mixtures under repeated crack movement.

The Overlay Test results in Figure 11 show a rapid reduction in normalized load during the early cycles, followed by a slower degradation stage after approximately 100–200 cycles. This indicates that most crack-opening damage developed during the initial loading stage, while the later cycles reflected the residual load-carrying capacity of each mixture. The poor-gradation control mixture, M2 C-Gpoor, showed the weakest response, with normalized load decreasing to 0.574 at 10 cycles and 0.226 at 1000 cycles. The standard-gradation control, M1 C-Gstd, retained slightly higher load at early cycles, while the SBS-only poor-gradation mixture, M3 S4.5-Si0-Gpoor, showed a modest improvement, reaching 0.628 at 10 cycles and 0.238 at 1000 cycles. This confirms that SBS improved binder cohesion but provided only limited resistance to repeated crack movement under poor gradation.

The optimized SBS–silane mixtures showed clearly better load retention. M4 S4.5-Si0.10-Gpoor retained normalized loads of 0.762 at 10 cycles and 0.250 at 1000 cycles, while M5 S4.5-Si0.10-Gstd showed the best response, with 0.816 at 10 cycles and 0.258 at 1000 cycles. Compared with M2, the improvements at 10 cycles were approximately 32.8% for M4 and 42.2% for M5, showing that the optimized SBS–silane system was particularly effective in delaying early crack-related damage. The performance ranking was therefore M5 > M4 > M3 ≈ M1 > M2, consistent with the HWT and SCB results. Overall, the OT results confirm that the optimized system improved crack-movement resistance mainly by enhancing load retention under cyclic displacement, with silane contributing to stronger aggregate–binder bonding and standard gradation providing additional structural stability.

### 3.8. Stage 2: Supplementary Freeze–Thaw Durability Evaluation of Optimized Mixtures

To further examine the durability of the optimized SBS–silane WMA system under environmental conditioning, supplementary freeze–thaw testing was conducted on the two best-performing mixtures: S4.5-Si0.10-Gpoor and S4.5-Si0.10-Gstd. These mixtures were selected because they represent the optimized SBS–silane combination under poor and standard gradation conditions, respectively. Due to the limited specimen availability and testing duration, the freeze–thaw evaluation was focused only on these two representative mixtures rather than on the full mixture matrix. The specimens were subjected to 10 freeze–thaw cycles before being evaluated using Hamburg Wheel Tracking and SCB tests.

**Figure 12:**
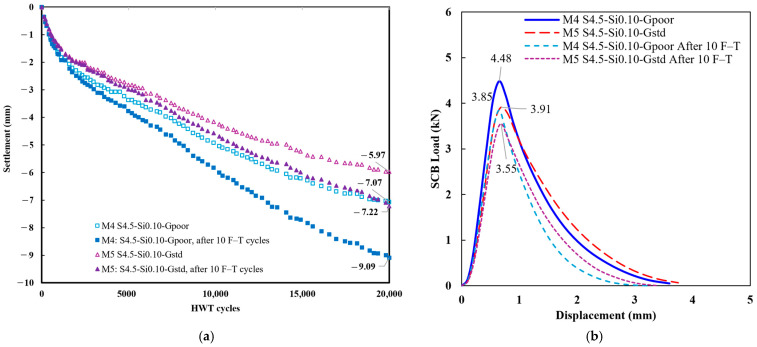
Effect of 10 freeze–thaw cycles on the (**a**) HWT settlement and (**b**) SCB load–displacement response of optimized SBS–silane WMA mixtures.

Figure 12a shows the HWT settlement curves before and after freeze–thaw conditioning. After 10 freeze–thaw cycles, both mixtures showed increased settlement, confirming that environmental conditioning reduced moisture-related deformation resistance. For S4.5-Si0.10-Gpoor, the final settlement increased from −7.07 mm to −9.09 mm. For S4.5-Si0.10-Gstd, the final settlement increased from −5.97 mm to −7.22 mm. Although both mixtures were affected by freeze–thaw conditioning, the standard-gradation mixture still showed lower final settlement than the poor-gradation mixture. This indicates that the optimized SBS–silane system performed better when supported by a more stable aggregate skeleton.

Figure 12b presents the SCB load–displacement responses before and after freeze–thaw conditioning. The peak load decreased from 4.48 kN to 3.85 kN for S4.5-Si0.10-Gpoor and from 3.91 kN to 3.55 kN for S4.5-Si0.10-Gstd. The reduction was more pronounced for the poor-gradation mixture, which also showed a faster post-peak load decay after freeze–thaw conditioning. In contrast, the standard-gradation mixture retained a more gradual post-peak response, indicating better fracture tolerance after environmental conditioning.

The supplementary freeze–thaw results confirm that environmental conditioning can reduce both rutting/moisture resistance and fracture resistance of the optimized SBS–silane WMA mixtures. However, the S4.5-Si0.10-Gstd mixture maintained better performance after 10 freeze–thaw cycles, while the S4.5-Si0.10-Gpoor mixture showed greater sensitivity to freeze–thaw-induced damage. These results further support the importance of combining SBS–silane modification with adequate aggregate gradation control for hot and humid pavement applications.

### 3.9. Performance-Adjusted Cost-Efficiency Assessment

**Figure 13:**
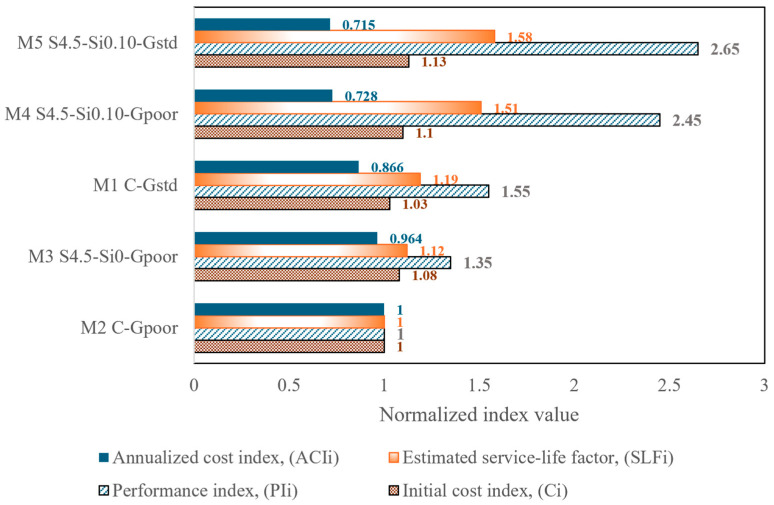
Scenario-based performance-adjusted cost and durability indices of selected WMA mixtures.

A scenario-based performance-adjusted cost-efficiency assessment was conducted to link laboratory performance with relative economic efficiency, as shown in Figure 13. The poor-gradation control mixture, M2 C-Gpoor, was used as the reference condition, with all indices normalized to 1.00. This assessment was not intended as a project-specific life-cycle cost analysis, but as a comparative index considering initial cost, performance improvement, estimated service-life factor, and annualized cost.

The SBS-only poor-gradation mixture, M3 S4.5-Si0-Gpoor, had a higher initial cost index of 1.08 and a performance index of 1.35. However, because its durability improvement was limited by moisture sensitivity and post-peak fracture loss, its annualized cost index decreased only slightly to 0.964, corresponding to a 3.6% cost saving. The standard-gradation control mixture, M1 C-Gstd, performed better, with a performance index of 1.55 and an annualized cost index of 0.866, giving a 13.4% cost saving. This shows that gradation improvement alone can provide a meaningful cost-efficiency benefit.

The optimized SBS–silane mixtures showed the strongest cost-efficiency response. For M4 S4.5-Si0.10-Gpoor, the initial cost index increased to 1.10, but the performance index reached 2.45, reducing the annualized cost index to 0.728 and giving a 27.2% saving relative to M2. The best result was obtained for M5 S4.5-Si0.10-Gstd, with an initial cost index of 1.13, a performance index of 2.65, an annualized cost index of 0.715, and a cost saving of 28.5%. The small difference between M4 and M5 suggests that the optimized SBS–silane system provided the main economic benefit, while standard gradation offered additional improvement. Overall, the results indicate that the higher initial material cost of the SBS–silane WMA system can be offset by improved durability when performance-adjusted service life is considered.

### 3.10. Discussions

#### 3.10.1. Research Limitations

The present study primarily focused on mixture-level performance evaluation of SBS–silane WMA under poor gradation conditions. Therefore, MSCR was selected as the primary binder-level rheological test because it provides a practical indicator of high-temperature deformation resistance within the scope of the current research stage and available project resources. Additional rheological and physicochemical investigations, including frequency sweep analysis, phase angle, G*/sinδ characterization, storage stability assessment, and RTFO/PAV aging evaluation, are recommended for future work to further clarify the long-term rheological stability and aging-related behavior of the SBS–silane modified binder system.

The present study mainly interpreted the adhesion-related improvement through mixture-level performance indicators such as wet ITS, TSR, and Hamburg Wheel Tracking response. Although these results consistently indicated improved moisture resistance after silane modification, direct chemical and microstructural investigations were outside the scope of the current research stage. Therefore, future studies are recommended to include surface free energy analysis, FTIR characterization, SEM observation, and adhesion energy measurements to further clarify the interfacial interaction mechanism between the SBS–silane modified binder and aggregate surface under moisture exposure.

Although the present study confirmed the benefit of silane through mixture-level moisture resistance and durability tests, direct adhesion measurements, such as surface free energy testing, were not conducted. Future work should include surface free energy analysis to quantify the asphalt–aggregate adhesion and debonding energy more directly, and to further clarify the role of silane in SBS-modified WMA mixtures.

Although the present study mainly focused on laboratory-scale performance evaluation, several durability-related tests were included, such as 20,000-cycle Hamburg Wheel Tracking under 60 °C water conditioning, Semi-Circular Bending, and Overlay Test analyses. These procedures were intended to simulate accelerated moisture–temperature exposure, rutting susceptibility, fracture resistance, and repeated crack-movement conditions. Nevertheless, additional long-term durability investigations, including oxidative aging, freeze–thaw cycling, and fatigue-related aging evaluation, are recommended for future studies to further verify the long-term field applicability of SBS–silane WMA systems under hot and humid environmental conditions.

#### 3.10.2. General Discussions

Overall, the results show that the performance of the SBS–silane WMA system was controlled by a balance between binder cohesion, interfacial adhesion, and aggregate gradation quality. SBS improved dry strength, Marshall stability, and deformation resistance, but the SBS-only mixtures still showed limited moisture resistance under poor gradation, confirming that binder reinforcement alone cannot fully prevent water-induced debonding. The addition of silane improved wet ITS and TSR, indicating better asphalt–aggregate bonding and strength retention after moisture exposure. This combined mechanism was further supported by the HWT, SCB, and Overlay Test results, where the optimized S4.5-Si0.10 mixture showed better rutting resistance, fracture tolerance, and crack-movement resistance than the control mixtures. The supplementary freeze–thaw results also confirmed that environmental conditioning reduced performance, but the standard-gradation optimized mixture retained lower HWT settlement and a more stable SCB post-peak response than the poor-gradation optimized mixture. Therefore, the main finding is not simply that SBS and silane improve WMA performance, but that durable WMA under hot and humid conditions requires the combined control of binder-phase cohesion, asphalt–aggregate adhesion, and aggregate skeleton quality.

## 4. Conclusions

This study developed an SBS–silane modified WMA system to improve moisture-induced debonding resistance under hot–humid conditions and marginal aggregate gradation. The results show that mixture durability was governed by the combined effects of binder cohesion, asphalt–aggregate interfacial bonding, and aggregate skeleton quality.

SBS modification increased dry strength, Marshall stability, and binder-level deformation resistance, confirming its role in improving binder cohesion and load-bearing capacity. However, SBS alone did not provide sufficient moisture protection: the TSR values of silane-free mixtures remained only 71.7–73.3%, despite the increase in dry ITS from 0.78 to 0.92 MPa and Marshall stability from 10.8 to 13.0 kN. This indicates that polymer reinforcement alone cannot fully address moisture-induced debonding when aggregate packing is poor.

The addition of silane shifted the improvement from strength gain to moisture-damage resistance. At 4.5% SBS, 0.10% silane increased wet ITS from 0.63 to 0.80 MPa and TSR from 73.3% to 85.1%, while Marshall stability increased from 12.3 to 13.8 kN. A higher silane dosage of 0.15% did not provide additional benefit, indicating that the optimum response depends on balanced interfacial modification rather than maximum additive content.

Gradation quality strongly affected moisture susceptibility. The control mixture showed a TSR reduction from 80.3% under standard gradation to 69.1% under poor gradation. In contrast, the optimized S4.5-Si0.10 system maintained TSR values of 88.9% and 85.1% under standard and poor gradation, respectively. This smaller performance loss confirms that silane improved the robustness of WMA against moisture exposure, although adequate aggregate packing remained important.

Advanced performance testing confirmed the practical benefit of the optimized system. Under poor gradation, the optimized SBS–silane mixture reduced Hamburg final settlement from −15.59 to −7.07 mm, corresponding to a 54.7% reduction. The SCB work of fracture increased from 1.34 to 5.20 J, and the Overlay Test load retention at 1000 cycles increased from 0.226 to 0.250. These results indicate that the optimized system improved resistance to rutting/stripping, fracture propagation, and repeated crack movement mainly through better post-peak and moisture-damage resistance rather than only higher peak strength.

The performance-adjusted cost assessment further suggested that the optimized mixture can be economically justified. Although S4.5-Si0.10-Gpoor had a higher initial cost index of 1.10, its improved performance reduced the annualized cost index to 0.728, equivalent to a 27.2% reduction relative to the poor-gradation control. This value should be interpreted as a scenario-based comparison rather than a project-specific life-cycle cost.

Overall, 4.5% SBS with 0.10% silane provided the most balanced WMA design in this study. The findings demonstrate that durable WMA for hot and humid regions should not rely only on polymer modification or anti-stripping treatment alone, but should combine binder-phase reinforcement, interfacial adhesion improvement, and aggregate gradation control. Future work should verify the proposed system under plant production, field construction, and long-term environmental aging conditions.

## Figures and Tables

**Table 1:** Basic properties of the base asphalt binder.

Property	Value	Test Method
Penetration at 25 °C, 0.1 mm	64	ASTM D5 [15]
Softening point, °C	49.5	ASTM D36 [16]
Ductility at 25 °C, cm	>100	ASTM D113 [17]
Rotational viscosity at 135 °C, Pa·s	0.46	ASTM D4402 [18]
Rotational viscosity at 165 °C, Pa·s	0.17	ASTM D4402 [18]
Specific gravity	1.03	ASTM D70 [19]
Flash point, °C	>260	ASTM D92 [20]

**Table 2:** Properties of SBS polymer modifier [3,21,22].

Property	Value
Polymer type	Styrene–butadiene–styrene
Physical form	Granule/pellet
Color	White to light yellow
Density, g/cm^3^	0.94
Styrene content, %	30
Volatile content, %	<1.0
Recommended mixing temperature, °C	165–175
Rotational viscosity of S4.5 binder at 135 °C, Pa·s	1.18
Rotational viscosity of S4.5 binder at 165 °C, Pa·s	0.42
Mixing time, min	45–60

**Table 3:** Properties of silane-based anti-stripping additive.

Property	Value
Chemical type	Amino-functional organosilane-based liquid additive
Main active chemistry	Hydrolysable alkoxy-silane groups with amine functionality
Physical form	Liquid
Color	Pale yellow to brown
General chemical structure	R–Si(OR′)_3_
Specific gravity at 25 °C	0.92
Viscosity at 25 °C, mPa·s	80
Flash point, °C	>100

**Table 4:** Basic properties of aggregates and mineral filler.

Material	Property	Value	Test Method
Coarse aggregate	Apparent specific gravity	2.68	ASTM C127 [23]
	Water absorption, %	0.85	ASTM C127 [23]
	Los Angeles abrasion loss, %	24.8	ASTM C131 [24]
	Flakiness index, %	13.5	ASTM D4791 [25]
Fine aggregate	Apparent specific gravity	2.63	ASTM C128 [26]
	Water absorption, %	1.25	ASTM C128 [26]
	Sand equivalent, %	58	ASTM D2419 [27]
Mineral filler	Specific gravity	2.72	ASTM C128 [26]
	Passing 0.075 mm sieve, %	96	ASTM C128 [26]

**Table 5:** Volumetric comparison of field core samples from conventional pavement and maintenance overlay using poor aggregate.

Parameter	Conventional Original Pavement Core	Maintenance Overlay Core Using Poor Aggregate
Number of field cores	3	3
Nominal maximum aggregate size, mm	12.5	12.5
Asphalt binder content, %	5.32 ± 0.08	5.38 ± 0.11
Bulk specific gravity, Gmb	2.421 ± 0.012	2.356 ± 0.018
Maximum specific gravity, Gmm	2.527 ± 0.009	2.536 ± 0.010
Air voids, Va, %	4.2 ± 0.4	7.1 ± 0.6
VMA, %	15.2 ± 0.5	13.4 ± 0.6
VFA, %	72.4 ± 2.1	47.0 ± 3.4

**Table 6:** Binder systems used in this study.

Binder ID	Base Binder (%)	SBS Content (% by Binder Mass)	Silane Content (% by Binder Mass)
B0 Control	100	0	0
S4.0-Si0	100	4.0	0
S4.0-Si0.05	100	4.0	0.05
S4.0-Si0.10	100	4.0	0.10
S4.0-Si0.15	100	4.0	0.15
S4.5-Si0	100	4.5	0
S4.5-Si0.05	100	4.5	0.05
S4.5-Si0.10	100	4.5	0.10
S4.5-Si0.15	100	4.5	0.15
S5.0-Si0	100	5.0	0
S5.0-Si0.05	100	5.0	0.05
S5.0-Si0.10	100	5.0	0.10
S5.0-Si0.15	100	5.0	0.15

**Table 7:** Main mixture design parameters.

Parameter	Value
Mixture type	Dense-graded WMA
Nominal maximum aggregate size, mm	12.5
Total binder content, % by mixture mass	5.4
Mineral filler content, %	5.0
WMA mixing temperature, °C	135
WMA compaction temperature, °C	125
SBS contents, % by binder mass	4.0, 4.5, 5.0
Silane contents, % by binder mass	0, 0.05, 0.10, 0.15
Gradation conditions	Standard, poor

**Table 8:** Binder preparation procedure.

Step	Material/Process	Temperature (°C)	Mixing Speed	Duration	Purpose
1	Heat-based binder	165 ± 5	—	60 min	Obtain fluid binder
2	Add SBS gradually	165 ± 5	3000 rpm	45 min	Disperse and swell SBS polymer
3	Homogenization	160 ± 5	500 rpm	20 min	Improve binder uniformity
4	Add silane additive	150 ± 5	500 rpm [11,13]	10 min	Incorporate anti-stripping additive
5	Short storage before mixing	150 ± 5	—	<2 h	Prevent excessive aging

**Table 9:** Mixture preparation conditions.

Parameter	Value
Mixture type	Dense-graded WMA
Nominal maximum aggregate size	12.5 mm
Total binder content	5.4% by mixture mass
Aggregate preheating temperature	145 ± 5 °C
Binder temperature before mixing	135 ± 5 °C
Target mixing temperature	135 ± 5 °C
Dry aggregate mixing time	30 s
Binder–aggregate mixing time	120 s
Filler mixing time	60 s
Total wet mixing time	180 s
Short-term conditioning before compaction	2 h at 135 °C

**Table 10:** Specimen compaction methods and target dimensions.

Test	Compaction Method	Specimen Size	Target Air Voids	Main Purpose
Marshall stability and flow	Marshall hammer	Ø101.6 × 63.5 mm	4.0 ± 0.5%	Basic strength and deformation
Dry ITS	Gyratory compactor	Ø100 × 63.5 mm	7.0 ± 0.5%	Tensile strength
Wet ITS/TSR	Gyratory compactor	Ø100 × 63.5 mm	7.0 ± 0.5%	Moisture susceptibility
Hamburg Wheel Tracking	Slab compactor/gyratory-cut slab	320 × 260 × 60 mm	7.0 ± 0.5%	Rutting and stripping resistance
SCB	Gyratory compacted cylinder, cut	Ø150 × 50 mm semicircle	7.0 ± 0.5%	Fracture response
Overlay Test	Slab/cut specimen	150 × 75 × 38 mm	7.0 ± 0.5%	Crack movement resistance

**Table 11:** Compaction and curing conditions.

Item	Condition
Marshall compaction effort	75 blows per side
Gyratory compaction angle	1.25°
Gyratory pressure	600 kPa
Gyratory speed	30 rpm
Target air voids for performance specimens	7.0 ± 0.5%
Target air voids for Marshall specimens	4.0 ± 0.5%
Cooling before demolding	24 h at room temperature
Cutting temperature	Room temperature
Storage before testing	25 ± 2 °C

## Data Availability

The data presented in this study are available within the manuscript. Additional data related to this work are available from the corresponding author upon reasonable request.
